# Features of the Molecular Structure and Luminescence of Rare-Earth Metal Complexes with Perfluorinated (Benzothiazolyl)phenolate Ligands

**DOI:** 10.3390/molecules24132376

**Published:** 2019-06-27

**Authors:** Tatyana V. Balashova, Mikhail E. Burin, Vasily A. Ilichev, Alyona A. Starikova, Alexey V. Marugin, Roman V. Rumyantcev, Georgy K. Fukin, Artem N. Yablonskiy, Boris A. Andreev, Mikhail N. Bochkarev

**Affiliations:** 1 G. A. Razuvaev Institute of Organometallic Chemistry of Russian Academy of Sciences, Tropinina street, 49, 60313 Nizhny Novgorod, Russia; 2 Institute of Physical and Organic Chemistry, Southern Federal University, Stachka avenue 194/2, 344090 Rostov-on-Don, Russia; 3 Nizhny Novgorod State University, Gagarina avenue 23/2, 603950 Nizhny Novgorod, Russia; 4 Institute for Physics of Microstructures of Russian Academy of Sciences, Akademicheskaya street 7, 603950 Nizhny Novgorod, Russia

**Keywords:** lanthanides, photoluminescence (PL), fluorinated benzothiazolate ligands, excited state lifetime

## Abstract

A set of Sc, Nd, Sm, Eu, Ho, Gd, Er, Yb complexes with perfluorinated 2-(benzothiazol-2-yl)phenolate ligands Ln(SON^F^)_3_(DME) were synthesized by the reactions of silylamides Ln[N(SiMe_3_)_2_]_3_ with phenol H(SON^F^). The structure of the initial phenol, Sc, and Er complexes was established using X-ray analysis, which revealed that the obtained compounds are mononuclear, in contrast to the binuclear non-fluorinated analogues [Ln(SON)_3_]_2_ synthesized earlier. All the obtained complexes, both in solid state and in tetrahydrofuran (THF) solutions, upon excitation by light with λ_ex_ 395 or 405 nm show intense luminance of the ligands at 440–470 nm. The Eu complex also exhibits weak metal-centered emission in the visible region, while the derivatives of Sm luminesces both in the visible and in the infrared region, and Nd, Er, and Yb complexes emit in the near IR (NIR) region of high intensity. DFT (density functional theory) calculation revealed that energy of frontier orbitals of the fluorinated complexes is lower than that of the non-fluorinated counterparts. The level of highest occupied molecular orbital (HOMO) decreases to a greater extent than the lowest occupied molecular orbital (LUMO) level.

## 1. Introduction

The search for new efficient photo- and electroluminophores still remains an urgent task. Near infrared (NIR) luminescence, especially from lanthanide complexes, such as Nd^3+^ and Yb^3+^, has emerged as an area of paramount interest due to its pioneering technological applications in fields ranging from bioimaging to optical communications [1,2,3,4,5,6,7]. However, since f-f transitions are largely forbidden, the ligand-free Ln^3+^ ions have very low molar absorption coefficients and hence the direct excitation of lanthanide ions always leads to modest or low luminescent intensities [8,9,10,11,12,13,14,15]. This problem can be solved with the help of organic ligands, which harvest excitation energy and transfer it to the attached Ln^3+^ ion, significantly increasing its luminescence (antenna effect) [16,17,18,19]. One of the most promising harvesting compounds is the benzothiazolate ligand (SON). The lanthanide complexes with SON ligands display excellent photo- and electroluminescence properties [20,21]. All these compounds, except for the scandium complex, have a dimeric structure, and all the ligands are bidentate [20,21]. However, the high energy of their triplet level does not coincide too much with the first excited state of NIR-luminescent ions Ln^3+^ [22]. As is known, the replacement of C–H bonds in a ligand with low energy C–F oscillators is able to lower the vibrational energy of the ligand and thereby enhances the luminescence intensity of the Ln^3+^ ions, especially for NIR emitting metals [23,24]. This effect is well manifested in Zn complexes with fluorinated benzothiazolate ligands, which show intense photoluminescence [25]. Following this approach, in search of new effective phosphors, we synthesized complexes of rare-earth metals based on perfluorinated benzothiazolate ligands (SON^F^) and studied their structure and photoluminescent (PL) properties.

## 2. Results and Discussion

### 2.1. Synthesis

**Scheme 1:**
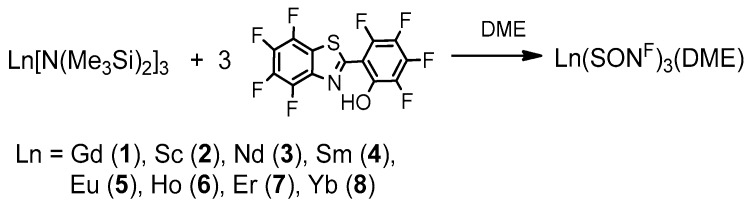
Synthetic scheme for complexes **1**–**8**.

The reactions of phenol H(SON^F^) with silylamides of rare-earth metals in a molar ratio of 3:1 (Scheme 1) occur readily at room temperature in a 1,2-dimetoxyethane (DME) solution and afford the expected products as light-yellow, yellow, or light-green finely crystalline powders in 65–78% yields.

**Figure 1:**
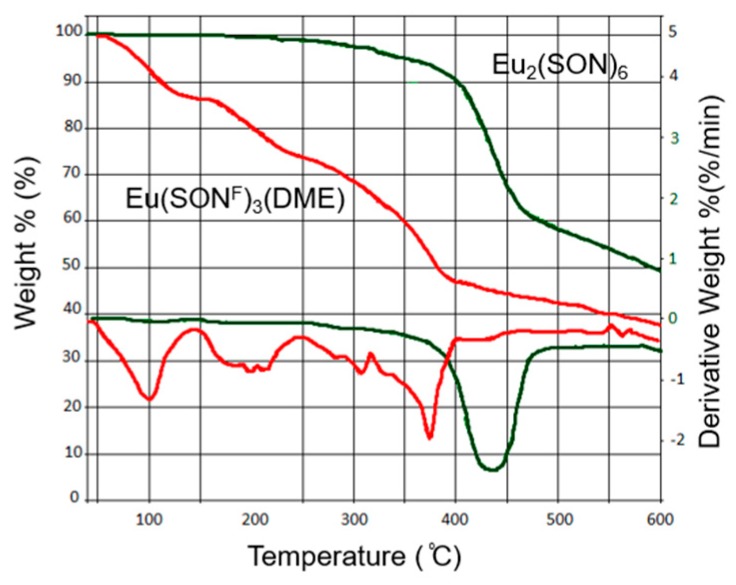
TGA of complexes Eu(SON^F^)_3_(DME)—red, and Eu_2_(SON)_6_—green.

A TGA study of the europium complex Eu(SON^F^)_3_(DME), as an example, showed that compounds with fluorinated ligands are less thermally stable than their non-fluorinated analogues (Figure 1).

### 2.2. Crystal Structures of H(SON^F^), ***2*** and ***7***

**Figure 2:**
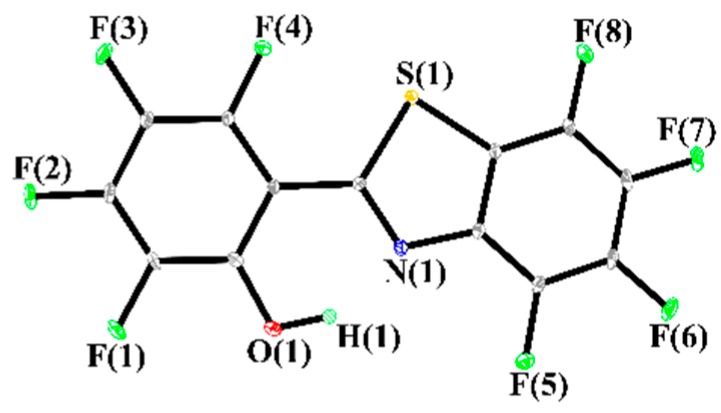
Molecular structure of independent molecules of H(SON^F^) (thermal ellipsoids are drawn at 30% probability).

According to the X-ray diffraction data, the asymmetric unit of the H(SON^F^) compound contains two disordered independent molecules, which have close geometric parameters. Molecules have a practically flat structure. The dihedral angles between benzothiazole and hydroxyphenyl fragments are 1.09–7.35°. This feature is in excellent agreement with previously published non-fluorinated analogues [26,27,28]. Such conformation, similar to non-fluorinated analogues [26,27,28,29], is stabilized by an intramolecular hydrogen bond O(1)H(1)···N(1) [1.71(2) Å] (Figure 2).

In a crystal, H(SON^F^) molecules form endless stacks with a frontal orientation (Figure S1). The dihedral angles between the planes of phenolate and five-membered heterocycles of neighboring ligands are 0.36–4.48°, and the distances between the centers of these fragments are 3.616–3.815 Å. These geometrical parameters are appropriate for the realization of intramolecular π···π interactions [29]. The angles between the molecules of the neighboring infinite stacks are 48.37–49.17° (Figure S1).

**Figure 3:**
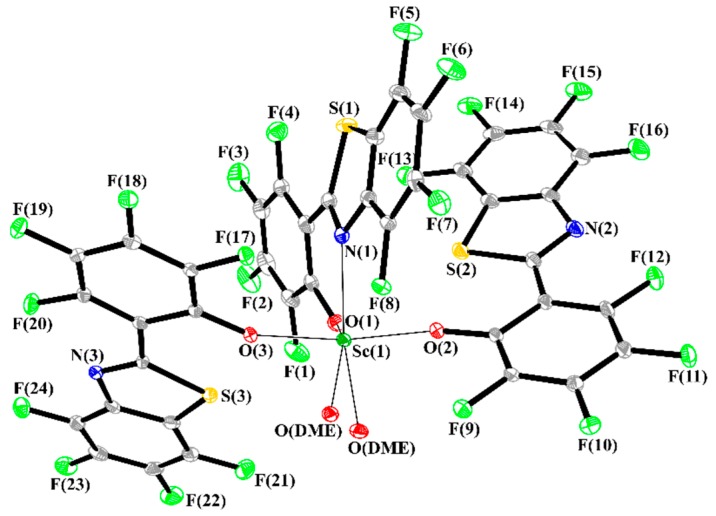
Molecular structure of **2** (thermal ellipsoids are drawn at 30% probability; DME molecule is presented only O-atoms for clarity). Selected distances (Å) and angles (°) in **2**: Sc(1)–O(3) 2.012(4), Sc(1)–O(1) 2.021(4), Sc(1)–O(2) 2.011(4), Sc(1)–O(4) 2.254(4), Sc(1)–O(5) 2.263(4), Sc(1)–N(1) 2.297(5), Sc(1)–F(8) 2.782; O(3)–Sc(1)–O(1) 90.12(16), O(3)–Sc(1)–O(2) 168.60(17), O(1)–Sc(1)–O(2) 94.97(17), O(3)–Sc(1)–N(1) 85.80(15), O(1)–Sc(1)–N(1) 78.66(17), O(2)–Sc(1)–N(1) 85.21(16).

According to X-ray analysis, complex **2** contains three SON^F^ anionic ligands and one neutral molecule, DME, coordinated to the Sc atom, which has a distorted pentagonal bipyramidal environment (Figure 3). It should be noted that two ligands in **2** are bonded to the Sc(1) atom monodentately via oxygen atoms. The sulfur atoms of the benzothiazolyl fragments in these ligands are directed towards the metal atom but do not interact coordinately. In turn, the third SON^F^ ligand coordinates to the Sc(1) atom bidentately via oxygen and nitrogen atoms. Such coordination was previously observed in a complex of yttrium with a 3-(2-benzothiazol-2-yl)-2-naphtholate ligand [30]. Analysis of Sc(1) coordination environment has shown the existence of the intramolecular Sc(1)···F(8) interaction, which is 2.782 Å (Figure 3).

This value lies in the range of strong interactions Ln···F [31] and evidently is a reason of the lowered thermostability of the complexes obtained. The calculation executed by the ToposPro program [32] confirms the Sc(1)···F(8) interaction and denotes the absence of Sc···S ones in **2**. The solid angle corresponding to the Sc(1)···F(8) interaction is 6.9%. Two monodentately coordinated SON^F^ ligands in **2** are practically flat. The dihedral angles between benzothiazole and hydroxyphenyl fragments are 2.30 and 6.89°. In turn, the planarity of the bidentately coordinated SON^F^ ligand is largely distorted (the same angle is 20.02°). The conformation of the SON^F^ ligand is typical for related complexes [21,30,33,34,35]. The planes of monodentately coordinated SON^F^ ligands of neighboring molecules are parallel to each other, which indicates the π···π interactions in crystal between these groups (Figure S2) [29].

**Figure 4:**
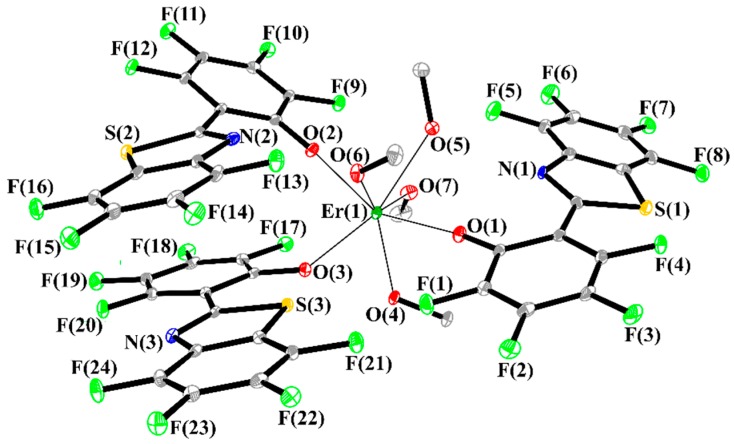
Molecular structure of **7** (thermal ellipsoids are drawn at 30% probability; hydrogen atoms are omitted for clarity). Selected distances (Å) and angles (°) in **7**: Er(1)–O(1) 2.177(2), Er(1)–O(2) 2.1782(19), Er(1)–O(3) 2.226(2), Er(1)–F(17) 3.107; O(1)–Er(1)–O(2) 147.51(8), O(1)–Er(1)–O(3) 115.32(8), O(2)–Er(1)–O(3) 85.20(8).

In complex **7**, the erbium atom is coordinated by three monodentate SON^F^ anionic ligands and four neutral methanol molecules (Figure 4). As in complex **2**, in the molecule of **7** there are shortened Ln···F contacts (Er(1)···F(17) 3.107 Å), which decrease its thermostability. According to calculations executed by the ToposPro program [32], the solid angle of this interaction is 3.4%. The coordination environment of the Er(1) cation is a distorted square antiprism, CN is 8.

It should be pointed out that in complex **7** all SON^F^ ligands are coordinated with the metal atom monodentally. Moreover, the ligands in the molecule have a different orientation: one of them is located in such a way that the nitrogen atom of the thiazole ring is directed to the metal atom, but the other two ligands are rotated so that the sulfur atom is closer to the Ln center. The monodentate coordination mode, observed as well in complex **2**, is unusual for non-fluorinated bensothiasolates and similar complexes. The difference in the structure of fluorinated and non-fluorinated compounds is explained evidently by the coordination between the Ln ion and the fluorine atoms, which occupy the coordination sphere of the metal atom and interfere with the coordination of the N and S atoms. Er···S (O, N) distances in **7** exceed the corresponding values in **2,** which agrees well with the difference in the cationic radii of Sc^3+^ and Er^3+^ [36]. Molecules of complex **7** in a crystal form dimeric pairs (Figure S3). The inter- and intramolecular distances between centers of aromatic systems of SON^F^ ligands are 3.483 Å and 3.482–3.758 Å, respectively, which indicates the existence of π···π interactions [29]. Besides this, in a crystal there is intermolecular N···H interactions (2.226 Å) (R(N)_vdw_ = 1.60 Å, R(H)_vdw_ = 1.01 Å) (Figure S3) [37].

### 2.3. Absorption Spectra and Judd-Ofelt Analysis

**Figure 5:**
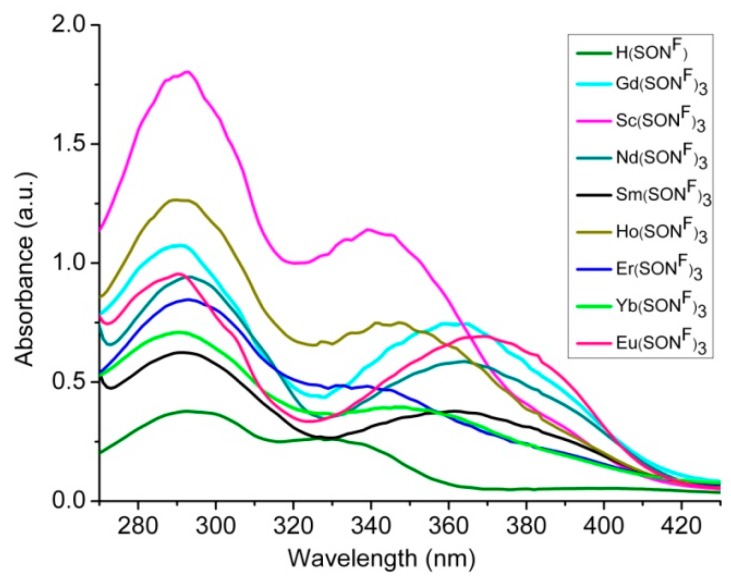
Absorption spectra of ligand H(SON^F^) and complexes **1**–**8** in THF solutions.

It is known that 2-(2-hydroxyphenyl)benzothiazole and respective benzothiazolate ligands can exist in enol and keto forms, which affects their molecular structure and electronic absorption spectra [34,38,39]. This feature is also inherent in perfluorated analogues, which is confirmed by their electronic absorption spectra in THF solutions. The spectra consist of three bands in the regions 250–310, 320–370, and 380–420 nm (Figure 5) and are assigned to intraligand π–π* transitions. Their relative intensity reflects the ratio of tautomeric forms in the compounds [34,39]. The spectra of complexes also contain two bands caused by intramolecular π–π* transitions. The observed bathochromic shift of the long-wavelength band in the spectra of the Nd, Sm, Eu, and Gd complexes compared to the Sc, Ho, Er, and Yb spectra is probably explained by some changes in the geometry of the coordination environment of metal ions upon transition from small to large Ln^3+^ ions.

**Figure 6:**
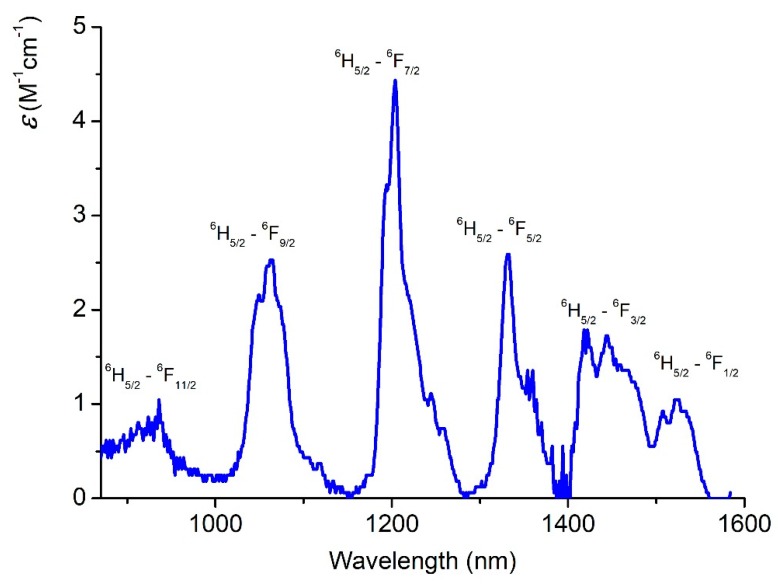
NIR absorption spectrum of complex **4** in THF solution.

To evaluate the calculated luminescent lifetime of the obtained complexes using the Judd-Ofelt theory, the spectrum of the solutions of compound **4** in THF in the NIR region was recorded using the compensatory subtraction method [40] (Figure 6). The spectrum consisted of six peaks assigned to f-f transitions from the ground ^6^H_5/2_ state to the excited ^6^F_J_ (J = 11/2, 9/2, 7/2, 5/2, 3/2, 1/2) and ^6^H_15/2_ energy levels (transitions from ^6^H_5/2_ to states ^6^F_3/2_ and ^6^H_15/2_ are combined in a single spectral peak).

Based on six absorption bands in the NIR region, the experimental values of line strength for each band were determined. Statistical analysis of the absorption spectra using the regression method yielded the Judd-Ofelt intensity parameters Ω_2_, Ω_4_, and Ω_6_, which indicate the intensity of metal-centered emission in the presence of a ligand. The values were found to be Ω_2_ ≈ 1.838, Ω_4_ ≈ 1.792, and Ω_6_ ≈ 2.320 (×10^−20^) cm^2^ with the error RMS Error = 0.115 × 10^−20^ cm^2^. Similar estimates of Judd-Ofelt parameters Ω_2_, Ω_4_, and Ω_6_ indicate an approximately equal contribution to the interaction from the effects stemming from the local symmetry around the lanthanide ion, so-named long range effects, and the rigidity of system, respectively. Using the calculated parameters Ω_i_, the strength of the electric dipole lines S_ED_ and the probabilities of spontaneous emission A_ED_ for all the observed optical transitions Sm^3+^ in **4** was made. The data for the most significant luminescent transitions summarized in Table 1 include the calculated A_ED_ values for the ^4^G_5/2_→X transitions in complex **4** in the THF solution, observed in the visible and NIR spectral regions, as well as the A_MD_ calculations based on the literature values for the strength of S_MD_ magnetic dipole lines in the luminescence spectrum [41,42]. The total luminescence probability A_∑_ = A_ED_ + A_MD_ and the branching ratio β for a given state are also provided.

The total luminescence probability A_Σ_ consists of two parts: the A_ED_, the electrodipole component calculated by the standard method using the obtained Ω_i_ values, and A_MD_, the magnetic-dipole component, with a non-zero value for a number of transitions, taking into account the selection rules typical for this class of compounds [43]. It should be noted that the probability values of magnetic dipole transitions do not depend on the matrix in Ln^3+^ compounds and can be taken from the literature. The values of the obtained intensity parameters Ω_2_–Ω_6_ allow the estimation of the total probability of all radiative transitions from the excited ^4^G_5/2_ state as A_rad_ ≈ 142 s^−1^ and the lifetime of this state as τ_calc_ ≈ 7.03 ms. Such values for the radiative lifetime of the excited state exceed by about two orders of magnitude of the experimental values of the lifetime, obtained from the excitation decay exponential curve, obtained by the method of time-resolved spectroscopy. The significant discrepancy between the experimental and calculated values of τ is apparently explained by the fact that the calculation method does not make it possible to take into account the nonradiative transitions [40,41,42].

### 2.4. Luminescence

**Figure 7:**
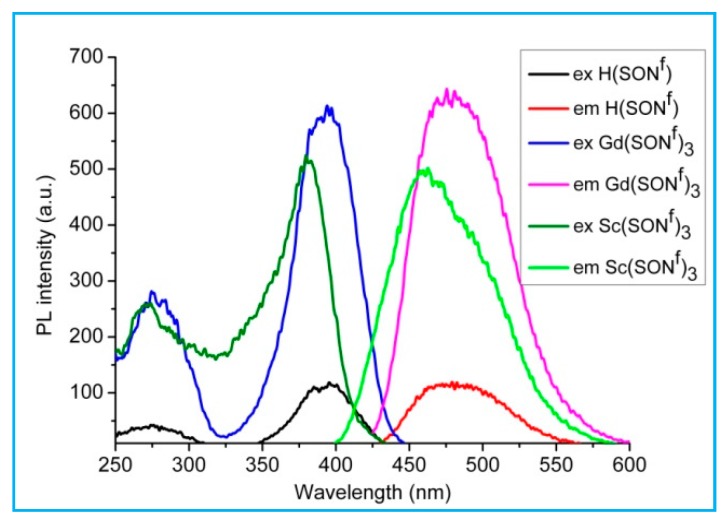
PL excitation and PL spectra of (benzothiazolyl)phenole H(SON^F^) and complexes **1** and **2** (λ_exc_ = 395 nm) in a THF solution (10^−5^ M).

It was previously shown that in the fluorescent spectra (PL) of non-fluorinated benzothiazolyl lanthanide complexes there are two bands: at around 430 nm (enol) and 520 nm (keto) forms [34]. Upon photo excitation with λ_exc_ 380–395 nm, the fluorinated complexes **1**–**2** and benzothiazole H(SON^F^) in THF or MeCN solutions exhibit only one broadened band in the region 460–480 nm, corresponding to the enol form, which, apparently, is more preferable in this case (Figure 7). The relative fluorescence quantum yield of the obtained compounds is close to 100%. This is significantly higher than that of non-fluorinated analogues [34].

**Figure 8:**
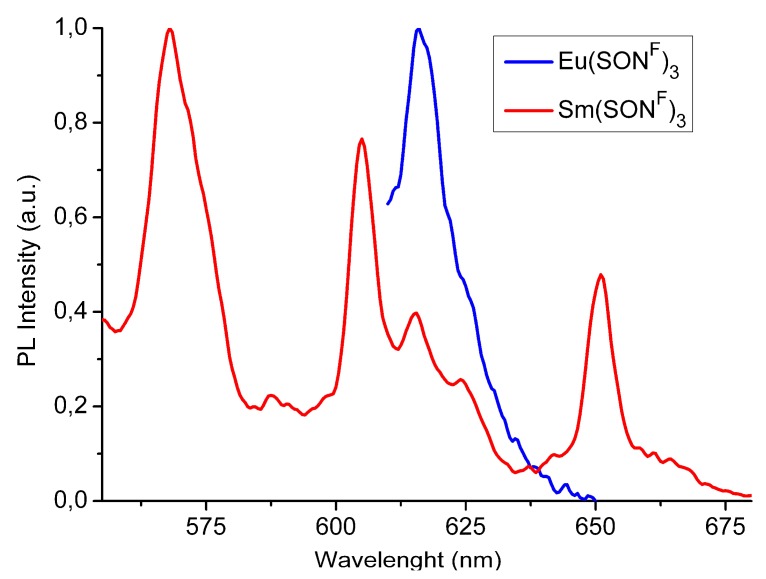
Normalized PL spectra of the complexes **4** and **5** (λ_exc_ = 395 nm) in a THF solution (10^−4^ M).

The T_1_ level of the SON^F^ ligand equal to 20,060 cm^−1^ was determined by the low-temperature photoluminescence spectrum of the gadolinium complex (Figure S4), by the position of the short-wave edge of the phosphorescence band, which is taken as the value corresponding to the 0–0 transition [44,45]. This position of the triplet level suggests that ligands can be an effective sensitizer for the PL of Ln^3+^ ions emitting in the visible region. According to this assumption, it has been found that the PL spectra of the Sm and Eu complexes, along with the bands of SON^F^ ligands, display the bands of the respective metal (Figure 8).

**Figure 9:**
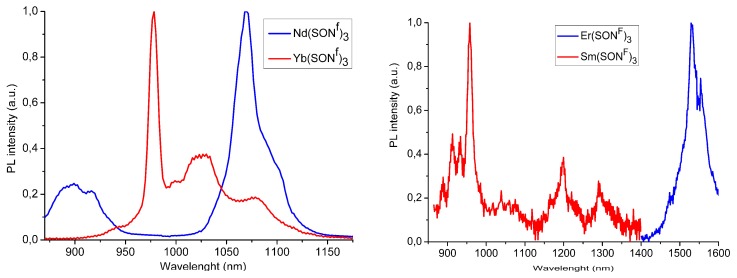
Normalized PL spectra of the solid samples of the complexes **3**, **4**, **7**, and **8** (λ_exc_ = 405 nm).

The spectrum of complex **4** in the visible region both in the solid state and in solution contains metal-centered emission bands characteristic for Sm^3+^, corresponding to transitions ^4^G_5/2_→^6^H_5/2_, ^6^H_7/2_, ^6^H_9/2_, and ^6^H_11/2_. As well the luminescence in the NIR region is caused by ^4^G_5/2_→^6^H_11/2_, ^6^H_13/2_, ^6^F_1/2_, ^6^F_3/2_, ^6^H_15/2_, ^6^F_5/2_, and ^6^F_11/2_ transition and Stark splitting (Figure 9). The measurement of the ^4^G_5/2_ excited state lifetime (τ_exp_ ≈ 15.5 μs) for complex **4** allowed the calculation of the PL quantum efficiency η = A_rad_/(A_rad_ + A_nonrad_) = τ_exp_/τ_calc_ (×100%) at room temperature, taking into account the above theoretically estimated radiative relaxation time (τ_calc_ ≈ 7.03 ms). The found value of η ≈ 0.22% corresponds to this parameter estimated for other Sm^3+^compounds [40,46] and can be considered evidence of the moderate luminescence ability of this class of compounds.

The europium complex **5**, in contrast to the non-emissive non-fluorinated analog, revealed a weak metal-centered luminescence in a THF solution at 616 nm, corresponding to the ^5^D_0_→^7^F_2_ transition (Figure 8). This observation is consistent with the known fact of quenching the luminescence of europium by phenol-substituted ligands due to the appearance in the complex of LMCT states and the low reduction potential of the Eu (III) ion [47]. The complexes Nd, Er, and Yb showed luminescence in the NIR region upon 405 nm laser excitation (Figure 9).

The triplet level of the ligands (20,600 cm^−1^) matches well the energy of the resonant level ^4^G_5/2_ of the Nd^3+^ ion (17135 cm^−1^), which provides the effective transfer of the excitation energy from the ligand to the metal and, as a result, leads to a high intensity of metal-centered emission. Effective sensitization can also be due to the short non-radiative relaxation time from the excited ^4^G_5/2_ state to the emission state ^4^F_3/2_ of the neodymium ion, which follows from the analysis of time-resolved spectroscopy data. It should be noted that the intensity of metal-centered emission in the PL spectra of the solid samples of complex **3** substantially exceeds the similar characteristic of that of non-fluoroinated analogues [21].

The difference between the energies of the triplet level of the ligand and the energy of the ^4^S_3/2_ level of erbium (2200 cm^−1^) does not quite match the optimal value of the energy gap (2500–3500 cm^−1^) necessary for effective sensitization. As a result, the Er complex **7** displayed only weak metal-centered emission at 1540 nm, corresponding to the ^4^I_13/2_ → ^4^I_15/2_ transition (Figure 9). In the case of the Yb complex **8,** such a way of energy transfer is impossible because of the too large energy gap between the triplet level of SON^F^ and the ^2^F_5/2_ resonant state of the Yb ion (10,330 cm^−1^). However, complex **8** gives a characteristic band of the ^2^F_5/2_ → ^2^F_7/2_ transition of the Yb^3+^ ion at 978 nm of moderate intensity, accompanied by Stark splitting bands in the region 1000–1100 nm. The sensitization of metal-centered PL in the Yb complex probably occurs due to the realization of a redox mechanism involving the photoinduced intramolecular electron transfer, as it was previously suggested for the non-fluorinated analogue [48]. The Ho complex **6** exhibits only ligand luminescence.

The kinetic PL measurements of complexes **3**, **4**, and **7** revealed that the Nd, Sm, and Er derivatives show monoexponential decay curves of the metal-centered emissions, with lifetimes of 1.07, 15.5, and 2 μs, respectively. For the Yb complex **8**, the exponent function is not fitted well and the lifetime varies in the range 5–7 μs (Figure S5). The lifetimes of the NIR PL of the obtained complexes are essentially shorter than those of compounds of respective lanthanides with other perfluorinated ligands (30–700 μs) [49,50]. Apparently, this behavior is due to the presence of strong quenchers (coordinated DME molecules) in these complexes. As is pointed out above, removing from the complex the anionic or neutral ligands containing quenching C–H groups leads to an increase of the luminescence efficiency [51,52,53].

### 2.5. DFT Modeling

**Figure 10:**
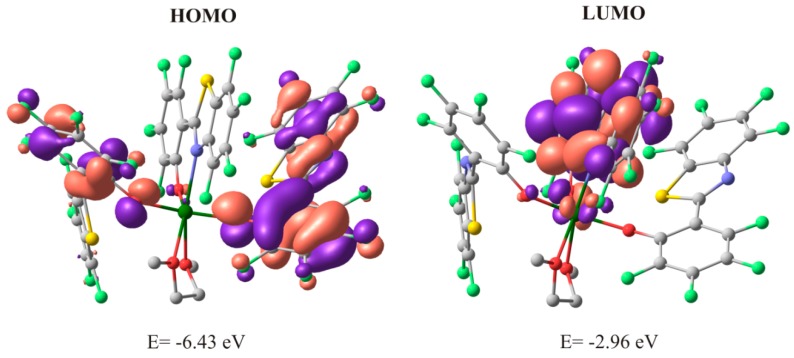
The shapes of HOMO and LUMO for complex **2** calculated by the DFT B3LYP/def2svp method (cutoff = 0.03 e/Å^3^).

To trace the difference in the electronic structure between fluorinated and non-fluorinated complexes, which can reflect on their luminescent characteristics, the DFT calculations of the scandium complex **2** were performed. The optimized geometry of **2** is given in Figure S6. The calculated parameters coincide with experimentally found values. The HOMO orbital is located on two ligands coordinated monodentately with the central ion. The HOMO of **2** represents an almost pure ligand orbital with a metal contribution of less than 1%, while the LUMO orbital is the almost pure orbital of the third ligand (bidentately bound) with the inclusion of a small contribution of the scandium atom (Figure 10). The HOMO–LUMO electronic transition can be considered, therefore, as an interligand charge transfer with a contribution of LMCT.

As in the case of similar complexes of zinc [25], the fluorination of ligands in complex **2** leads to a decrease in the energies of the frontier orbitals. However, in the scandium complex with the fluorinated ligand, a more significant decrease in HOMO and a less significant decrease in the LUMO in comparison with the non-fluorinated analogue occurs [20]. This can probably be explained by the different coordination of the ligands: in complex **2**, two ligands are coordinated monodentately and the third one bidentately, while in the non-fluoronated counterpart all three bensothiasolyl ligands are bound with Sc bidentately. Since the electron density of HOMO in complex **2** is located on a monodentately bound ligand, which is absent in the non-fluorinated compound, a difference arises in the energy of HOMO. The bidentately coordinated ligands, on which LUMO is mainly focused, the energy of LUMO in both complexes therefore decreases insignificantly. The absorption spectrum and photoluminescence of **2** is slightly red-shifted relative to the non-fluorinated analogue (Figure S6).

## 3. Materials and Methods

### 3.1. Characterization and Measurements

All manipulations were carried out under vacuum using standard Schlenk techniques. Solvents (DME and THF) were purified by distillation from sodium/benzophenone ketyl. The lanthanide complexes Ln[N(SiMe_3_)_2_]_3_ were synthesized according to the procedure published elsewhere [54]. 2-(3,4,5,6-Tetrafluoro-2-hydroxyphenyl)-4,5,6,7-tetrafluorobenzothiazole (H(SON^F^)) was synthesized by a multistep method, described in publications [25]. The molecular structure of the compound was established in this work. The C, H, and N elemental analyses were performed at the Elementar Vario EL cube Analyzer (Elementar Analysensysteme GmbH, Langenselbold, Germany). The lanthanide content was analyzed by complexometric titration. IR spectra were obtained on a Perkin Elmer 577 spectrometer (Waltham, MA, USA) as a Nujol mull on KBr plates in the 450–4000 cm^−1^ region. Samples were prepared as a Nujol mull on KBr plates. Emission spectra were registered from 300 to 700 nm on a fluorescent spectrometer Perkin Elmer LS-55. Relative PL quantum yields of the synthesized complexes in CH_3_CN were measured at ambient conditions with an excitation wavelength of 390 nm, and the values were calculated using Rhodamine 6G in ethanol (Φ = 0.95) as standard [55], according to the known procedure [56]. A Perkin Elmer Lambda 900 UV-vis absorption spectrophotometer was used to record absorption spectra. Registration of absorption and emission spectra was performed in a 1 cm fluorescent quartz cuvette. UV-Vis absorption spectra of samarium complex was recorded on a Shimadzu UV-3600 from 200 to 1600 nm. ^19^F-NMR spectra were recorded at 376 MHz in d8-THF by using an Agilent DD2 400 spectrometer (Agilent Technologies, Santa Clara, CA, USA). Deuterated solvent d8-THF was used as received from Aldrich (St. Louis, MO, USA).

### 3.2. Synthesis Details

#### 3.2.1. Synthesis of Gd(SON^F^)_3_(DME) (**1**)

A solution of 2-(3,4,5,6-tetrafluoro-2-hydroxyphenyl)-4,5,6,7-tetrafluorobenzothiazole (119 mg, 0.321 mmol) in DME (10 mL) was added to a solution of Gd[N(SiMe_3_)_2_]_3_ (68 mg, 0.107 mmol) in DME (5 mL). The reaction mixture was stirred for 30 min at room temperature. The solution was concentrated to 2 mL. The light-yellow precipitate of complex **1** had been landed from hexane, separated by decantation, washed with cold hexane, and dried in vacuum, yielding 100 mg (65%). Anal. Calcd. (%) for C_43_H_10_F_24_GdN_3_O_5_S_3_ (1357.96) C, 38.03; H, 0.74; Gd, 11.58; N, 3.09; S, 7.08. Found (%) C, 38.06; H, 0.75; Gd, 11.61; N, 3.04; S, 7.11. IR (KBr, ν, cm^−1^): 1650 (m), 1581 (w), 1256 (s), 1184 (m), 1099 (m), 1052 (m), 995 (s), 886 (w), 846 (m), 817 (w), 751 (m), 621 (w), 600 (w), 523 (w), 497 (w).

The complexes of Sc, Nd, Sm, Eu, Ho, Er, and Yb were obtained in a similar manner.

#### 3.2.2. Synthesis of Sc(SON^F^)_3_(DME) (**2**)

A total of 46 mg (75%) of **2** was prepared from 55 mg (0.148 mmol) of H(SON^F^) and 26 mg (0.049 mmol) of Sc[N(SiMe_3_)_2_]_3_. Anal. Calcd. (%) for C_43_H_10_F_24_N_3_O_5_S_3_Sc (1245.68) C, 41.46; H, 0.81; N, 3.37; S, 7.72; Sc, 3.61. Found (%) C, 41.38; H, 0.75; N, 3.34; S, 7.79; Sc, 3.54. ^19^F-NMR (376 MHz, THF-d8, 20 °C), δF: −140.5 (m), −141.9 (m), −149.0 (m), −157.1 (m), −160.4 (m), −161.9 (m), −163.5 (m), −179.0 (m).

The qualitative crystals suitable for X-ray analysis were obtained by recrystallization of the product from a mixture of DME–hexane.

#### 3.2.3. Synthesis of Nd(SON^F^)_3_(DME) (**3**)

Complex **3** was prepared from 94 mg (0.253 mmol) of H(SON^F^) and 53 mg (0.085 mmol) of Nd[N(SiMe_3_)_2_]_3_. Yield: 91 mg (75%). Anal. Calcd. (%) for C_43_H_10_F_24_N_3_NdO_5_S_3_ (1344.95) C, 38.40; H, 0.75; N, 3.12; Nd, 10.72; S, 7.15. Found (%) C, 38.39; H, 0.77; N, 3.09; Nd, 10.69; S, 7.12.

#### 3.2.4. Synthesis of Sm(SON^F^)_3_(DME) (**4**)

A total of 85 mg (76%) of **4** was obtained from 92 mg (0.248 mmol) of H(SON^F^) and 52 mg (0.082 mmol)of Sm[N(SiMe_3_)_2_]_3_. Anal. Calcd. (%) for C_43_H_10_F_24_N_3_O_5_S_3_Sm (1351.07) C, 38.23; H, 0.75; N, 3.11; S, 7.12; Sm, 11.13. Found (%) C, 38.20; H, 0.79; N, 3.05; S, 7.19; Sm, 11.11.

#### 3.2.5. Synthesis of Eu(SON^F^)_3_(DME) (**5**)

Complex 4 (83 mg, 73%) was isolated from the reaction of 93 mg (0.251 mmol) of H(SON^F^) with Sm[N(SiMe_3_)_2_]_3_ (53 mg, 0.084 mmol). Anal. Calcd. (%) for C_43_H_10_EuF_24_N_3_O_5_S_3_ (1352.67) C, 38.18; H, 0.75; Eu, 11.23; N, 3.11; S, 7.11. Found (%) C, 38.19; H, 0.76; Eu, 11.27; N, 3.08; S, 7.16.

#### 3.2.6. Synthesis of Ho(SON^F^)_3_(DME) (**6**)

A total of 86 mg (0.232 mmol) of H(SON^F^) and 50 mg (0.077 mmol) of Ho[N(SiMe_3_)_2_]_3_ yielded 74 mg (70%) of **6**. Anal. Calcd. (%) for C_43_H_10_F_24_HoN_3_O_5_S_3_ (1365.64) C, 37.82; H, 0.74; Ho, 12.08; N, 3.08; S, 7.04. Found (%) C, 37.83; H, 0.69; Ho, 12.05; N, 3.02; S, 7.07.

#### 3.2.7. Synthesis of Er(SON^F^)_3_(DME) (**7**)

Complex **7** (92 mg, 75%) was obtained from H(SON^F^) (100 mg, 0.269 mmol) and Er[N(SiMe_3_)_2_]_3_ (58 mg, 0.089 mmol). Anal. Calcd. (%) for C_43_H_10_ErF_24_N_3_O_5_S_3_ (1367.97) C, 37.75; H, 0.74; Er, 12.23; N, 3.07; S, 7.03. Found (%) C, 37.79; H, 0.76; Er, 12.21; N, 3.02; S, 7.05.

The crystals suitable for X-ray analysis were obtained by recrystallization of the product from the methanol.

#### 3.2.8. Synthesis of Yb(SON^F^)_3_(DME) (**8**)

From 107 mg (0.288 mmol) of H(SON^F^) and 63 mg (0.096 mmol) of Yb[N(SiMe_3_)_2_]_3_ 102 mg (77%) of **8** was prepared. Anal. Calcd. (%) for C_43_H_10_F_24_N_3_O_5_S_3_Yb (1373.76) C, 37.60; H, 0.73; N, 3.06; S, 7.00; Yb, 12.60. Found (%) C, 37.57; H, 0.75; N, 2.97; S, 7.03; Yb, 12.58.

The IR spectra of complexes **2**–**8** are identical to that of **1**.

### 3.3. X-Ray Single-Crystal Diffraction Analysis of H(SON^F^), ***2***, and ***7***

The X-ray diffraction data were collected on an Agilent Xcalibur E (**2**) and a Bruker D8 Quest (H(SON^F^) and **7**) diffractometers (MoK_α_ radiation, ω-scan technique, λ = 0.71073 Å, Bruker AXS, Madison, WI, USA). The intensity data were integrated by CrysAlisPro [57] for **2** and by SAINT [58] for H(SON^F^) and **7**. The structures H(SON^F^), **2**, and **7** were solved by dual method [59] and were refined on F^2^_hkl_ using the SHELXL package [60]. All non-hydrogen atoms were refined anisotropically. All hydrogen atoms were placed in calculated positions and were refined in the riding model. The H(1) in H(SON^F^) was found from the Fourier difference synthesis of electron density and refined isotropically. SCALE3 ABSPACK [61] for **2** and SADABS [62] for H(SON^F^) and **7** were used to perform area-detector scaling and absorption corrections. The main crystallographic data and structure refinement details for H(SON^F^), **2**, and **7** are presented in Table 2. Cambridge Crystallographic Data Centre (CCDC) 1,883,260 (H(SON^F^)), 1,883,261 (**2**), and 1,883,259 (**7**) contain the supplementary crystallographic data for this paper. These data can be obtained free of charge www.ccdc.cam.ac.uk/structures/from the Cambridge Crystallographic Data Centre.

### 3.4. Computational Details

Density functional theory (DFT) calculations were performed using the Gaussian 09 program package [63]. A full geometry optimization of complex **2** was accomplished at the B3LYP/def2svp level, with subsequent checking for the stability of the DFT wave function. Structural visualizations in Figure 10 were produced with the program suite ChemCraft [64].

### 3.5. Time-Resolved Spectroscopy

The PL decay times were measured under pulsed excitation with a Spectra-Physics optical parametric oscillator at 430 and 474 nm (Spectra-Physics, Santa Clara, CA, USA). The duration of the excitation pulse was 10 ns. The PL signal in the 450–800 nm range was dispersed using a grating spectrometer Acton-2300 (Teledyne Princeton Instruments, Trenton, NJ, USA) and detected using a Si-based photomultiplier tube (PMT) (Hamamatsu photonics, Hamamatsu, Japan) and a LeCroy digital oscilloscope (Teledyne LeCroy, Chestnut Ridge, NY, USA).

## 4. Conclusions

A set of rare-earth metals complexes with perfluorinated 2-(2-hydroxyphenyl)benzothiazolate ligands were obtained and structurally characterized. All the compounds (except Sc and Gd) upon excitation by UV light displayed a metal-centered PL of moderate intensity. The emission spectra corresponded to respective Ln^3+^ ions. The complexes of Sc and Gd exhibited only the PL of the ligands in a high quantum yield. The data obtained are consistent with the known theoretical approach, according to which the replacement of C–H groups in the ligands with C–F bonds results in an increase in the stability and luminescent activity of lanthanide emitters. The high luminescent activity of the Er, Yb, and especially Nd complexes provides a basis for testing these materials in organic light-emitting diodes (OLED) as electroluminescent layers, as well as in the detectors of ionizing radiation, which will be the subject of further research.

## Figures and Tables

**Table 1:** Parameters of luminescence of samarium complex **4** in THF solution.

^4^G_5/2_→^2S+1^L_J_	λ (nm)	S_ED_ (×10^−23^ cm^2^)	A_ED_ (s^−1^)	S_MD_ (×10^−23^ cm^2^)	A_MD_ (s^−1^)	A_Σ_ (s^−1^)	β (%)
^6^F_5/2_	958	16.3	5.56	6.62	2.48	8.04	5.65
^6^F_3/2_	933	2.2	0.81	8.46	3.44	4.25	2.99
^6^H_13/2_	795	4.5	2.69	0	0	2.69	1.89
^6^H_11/2_	706	14.4	12.28	0	0	12.28	8.63
^6^H_9/2_	650	37.2	40.73	0	0	40.73	28.63
^6^H_7/2_	605	36.2	49.04	5.81	8.66	57.70	40.56
^6^H_5/2_	568	1.62	2.65	5.73	10.32	12.97	9.12

**Table 2:** X-ray data collection and refinement parameters for H(SON^F^), **2**, and **7**.

Complex	H(SON^F^)	2	7
Empirical formula	C_13_HF_8_NOS	C_43_H_10_F_24_N_3_O_5_S_3_Sc	C_43_H_16_F_24_N_3_O_7_S_3_Er
Formula weight	371.21	1245.68	1406.03
T (K)	100(2)	100(2)	100(2)
Crystal system	Monoclinic	Triclinic	Triclinic
Space group	Cc	P-1	P-1
Unit cell dimensions			
a (Å)	9.4594(3)	11.1909(5)	12.0137(7)
b (Å)	12.5014(4)	14.0354(6)	12.1785(7)
c (Å)	20.1166(6)	15.0479(7)	16.3474(9)
α (°)	90	99.295(4)	84.9199(15)
β (°)	93.6280(10)	109.580(4)	88.0365(14)
γ (°)	90	94.136(4)	72.0830(13)
V (Å^3^)	2374.14(13)	2177.28(19)	2266.8(2)
Z	8	2	2
D_calc._ (g⋅cm^−3^)	2.077	1.900	2.060
Absorption coefficient, μ (mm^−1^)	0.386	0.470	2.146
F (000)	1456	1228	1366
Crystal size (mm^3^)	0.50 × 0.50 × 0.50	0.30 × 0.25 × 0.03	0.16 × 0.13 × 0.04
Range for data collection, θ (°)	2.029–32.000	2.931–25.000	2.502–30.034
Limiting indices	−14 ≤ h ≤ 14 −18 ≤ k ≤ 18 −29 ≤ l ≤ 29	−13 ≤ h ≤ 13 −16 ≤ k ≤ 16 −17 ≤ l ≤ 17	−16 ≤ h ≤ 16 −17 ≤ k ≤ 17 −23 ≤ l ≤ 23
Reflections collected/unique	17441/8202	21419/7655	32615/13211
R_(int)_	0.0199	0.0497	0.0504
Completeness (to θ)	99.9% (25.242)	99.6% (25.000)	99.8% (25.242)
Data/restraints/parameters	8202/184/591	7655/0/714	13211/13/750
Goodness-of-fit (GOF) on F^2^	1.024	1.020	1.031
Final R indices [I > 2σ(I)]	R_1_ = 0.0283 wR_2_ = 0.0755	R_1_ = 0.0716 wR_2_ = 0.1652	R_1_ = 0.0387 wR_2_ = 0.0708
R indices (all data)	R_1_ = 0.0305 wR_2_ = 0.0767	R_1_ = 0.1151 wR_2_ = 0.1827	R_1_ = 0.0555 wR_2_ = 0.0750
Largest diff. peak/hole (e Å^−3^)	0.478/−0.349	1.109/−0.451	1.859/−1.262

## Supplementary Materials

The following are available online. Figure S1: Packing motifs of H(SON^F^), Figure S2: Fragment of crystal packing of **2**. The F and H atoms are omitted for clarity, Figure S3: Fragment of crystal packing of **7**. The F and H atoms are omitted for clarity, Figure S4: Photoluminescence spectrum of the gadolinium complex **1** at 77 K, Figure S5: NIR PL kinetics of Er(SON^F^)_3_ at 1530, Nd(SON^F^)_3_ at 1070, Sm(SON^F^)_3_ at 645 and Yb(SON^F^)_3_ at 975 nm. Excitation – Nd:YAG laser with λ 355 nm (pulse duration ~5 ns). Red lines are fitting curves, Figure S6: The optimized geometry of **2**, Figure S7: Normalized EPL, PL and absorption spectra of nonfluorinated and fluorinated scandium complexes in THF solution (λ_exc_ = 385 nm, 10^−5^ M).
