# Supplementary material for: Features of the Molecular Structure and Luminescence of Rare-Earth Metal Complexes with Perfluorinated (Benzothiazolyl)phenolate Ligands
Source: Molecules. 2019 Jun 27;24(13):2376. doi: 10.3390/molecules24132376 (PMC6651842; doi:10.3390/molecules24132376)
Supplement: Supplementary file 1 [file molecules-24-02376-s001.pdf]

Supplementary Materials

Features of the Molecular Structure and Luminescence of Rare-Earth Metal Complexes with Perfluorinated (Benzothiazolyl)phenolate Ligands

Tatyana V. Balashova [1,*], Mikhail E. Burin [1], Vasily A. Ilichev [1], Alyona A. Starikova [2], Alexey V. Marugin [3], Roman V. Rumyantcev [1], Georgy K. Fukin [1], Artem N. Yablonskiy [4], Boris A. Andreev [4] and Mikhail N. Bochkarev [1,3,*]

1 G. A. Razuvaev Institute of Organometallic Chemistry of Russian Academy of Sciences, Tropinina street, 49, 60313 Nizhny Novgorod, Russian Federation; michail.burin@yandex.ru (M.E.B.); ilichev@iomc.ras.ru (V.A.I.); romanrum@iomc.ras.ru (R.V.R.); gera@iomc.ras.ru (G.K.F.)

2 Institute of Physical and Organic Chemistry, Southern Federal University, Stachka avenue 194/2, 344090 Rostov-on-Don, Russian Federation; alstar@ipoc.sfedu.ru

3 Nizhny Novgorod State University, Gagarina avenue 23/2, 603950 Nizhny Novgorod, Russian Federation; marugin@rf.unn.ru

4 Institute for Physics of Microstructures of Russian Academy of Sciences, Akademicheskaya street 7, 603950 Nizhny Novgorod, Russian Federation; yablonsk@ipm.sci-nnov.ru (A.N.Y.); boris@ipmras.ru (B.A.A.)

* Correspondence: petrovsk@iomc.ras.ru (T.V.B.); mboch@iomc.ras.ru (M.N.B.) Tel.: +7 (831) 435-4021 (M.N.B.)

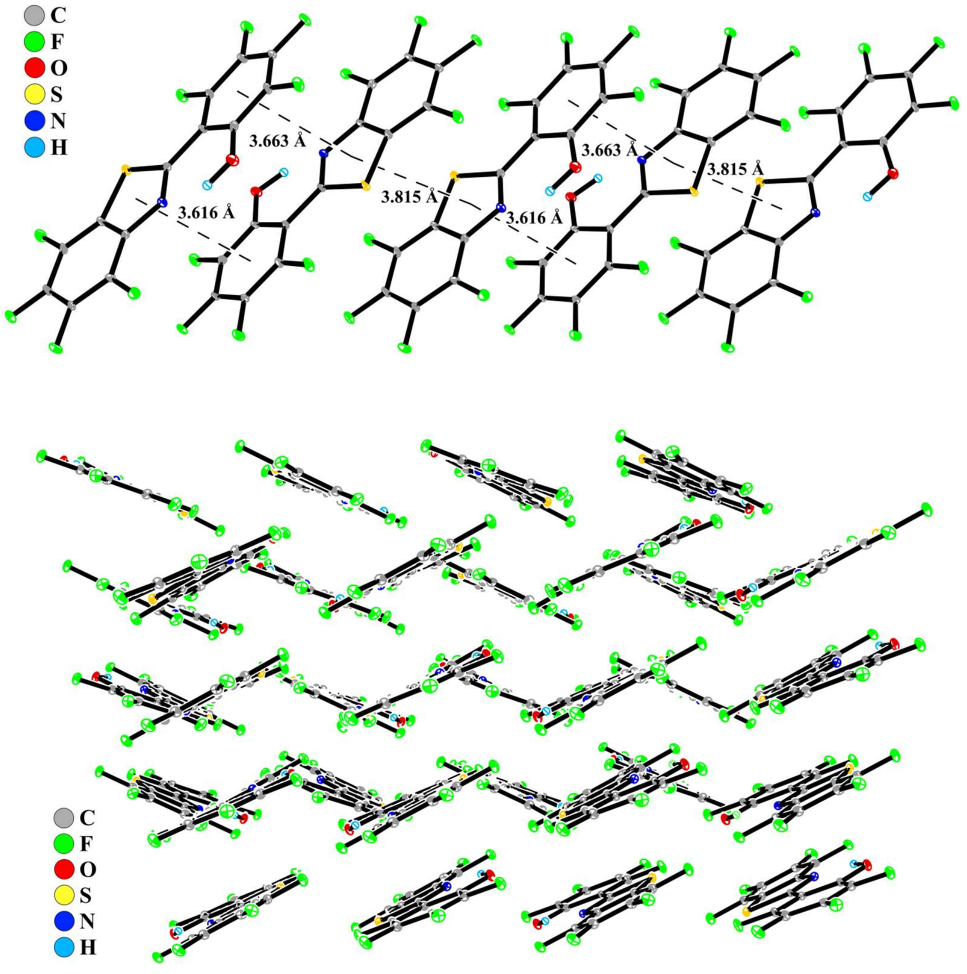

Figure S1. Packing motifs of H(SON^{F}).

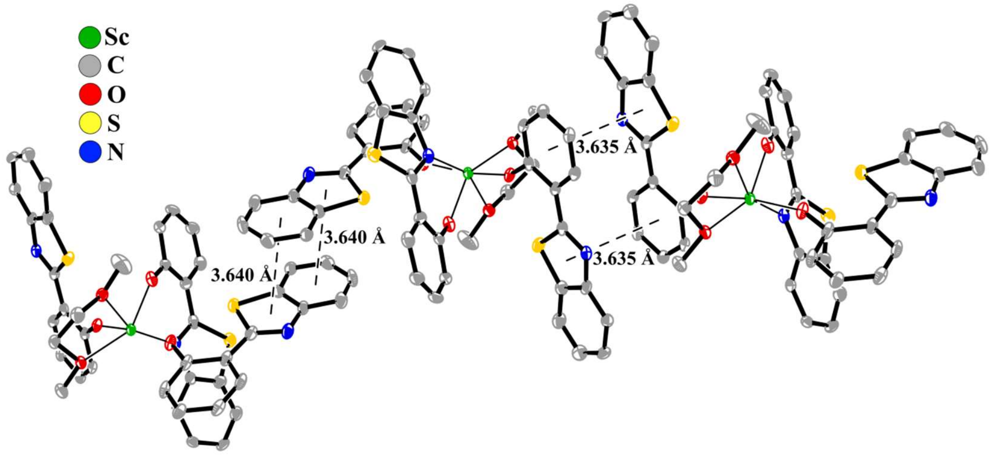

Figure S2. Fragment of crystal packing of **2**. The F and H atoms are omitted for clarity.

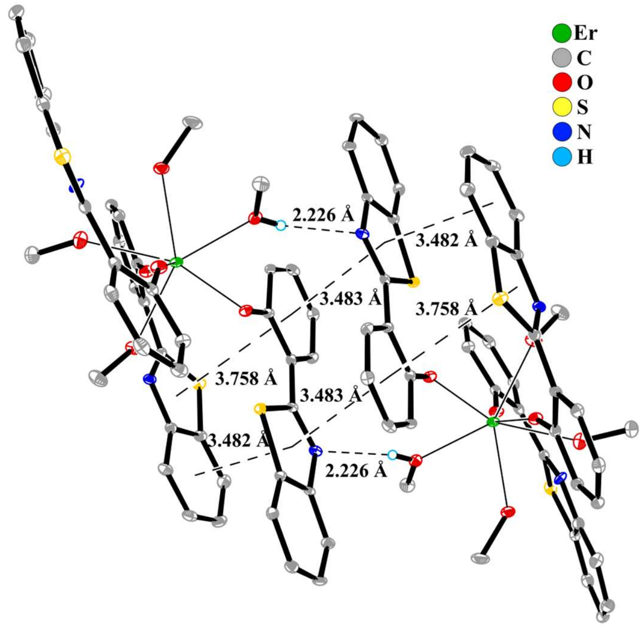

Figure S3. Fragment of crystal packing of **7**. The F and H atoms are omitted for clarity.

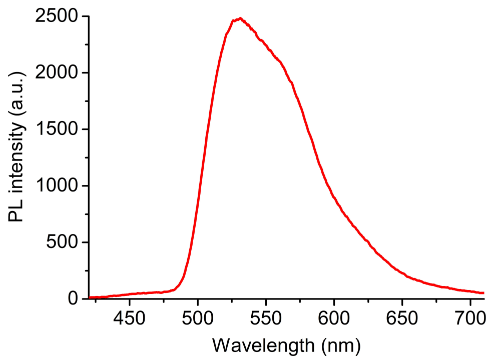

Figure S4. Photoluminescence spectrum of the gadolinium complex **1** at 77 K.

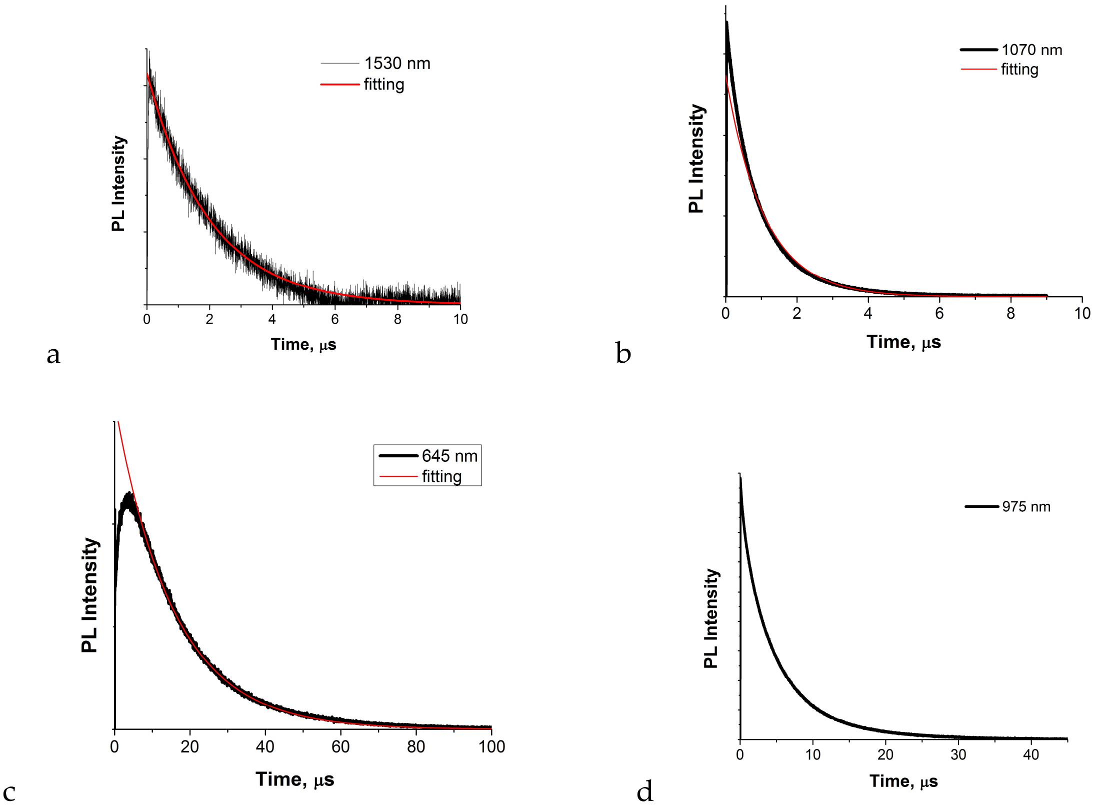

Figure S5. NIR PL kinetics of Er(SON^F)$_3$ at 1530 (**a**), Nd(SON^F)$_3$ at 1070 (**b**), Sm(SON^F)$_3$ at 645 (**c**) and Yb(SON^F)$_3$ at 975 nm (**d**). Excitation – Nd:YAG laser with λ 355 nm (pulse duration ~5 ns). Red lines are fitting curves.

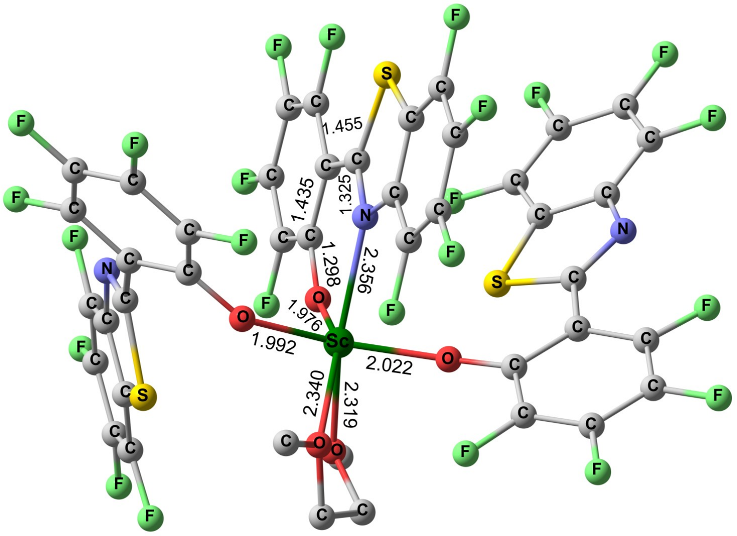

Figure S6. The optimized geometry of **2**.

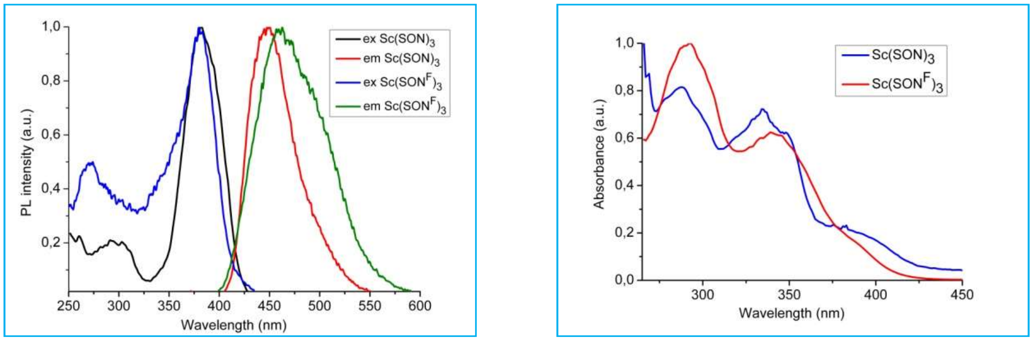

a **b**

Figure 7. Normalized EPL, PL (**a**) and absorption (**b**) spectra of nonfluorinated and fluorinated scandium complexes in THF solution (λ_{exc} = 385 nm, 10^{-5} M).
